# Comprehensive Management of Polycystic Ovary Syndrome: Effect of Pharmacotherapy, Lifestyle Modification, and Enhanced Adherence Counseling

**DOI:** 10.7759/cureus.35415

**Published:** 2023-02-24

**Authors:** Mugdha Jungari, Amruta Choudhary, Naresh Kumar Gill

**Affiliations:** 1 Obstetrics and Gynaecology, Datta Meghe Medical College, Datta Meghe Institute of Medical Sciences (Deemed to be University), Nagpur, IND; 2 Community Medicine, National Center for Vector Borne Diseases Control, Ministry of Health & Family Welfare, New Delhi, IND

**Keywords:** polycystic ovary syndrome (pcos), enhanced adherence counselling (eac), metformin, lifestyle modification (lsm), pcos

## Abstract

Introduction

Polycystic ovary syndrome (PCOS) is a common endocrine disorder in women of childbearing age in India, which often presents as menstrual irregularities, infertility, acanthosis nigricans, etc. Metabolic disturbances associated with PCOS predispose patients to cardiovascular diseases, which may be avoided by effective management. The aim of the current study was to evaluate the role of lifestyle modification (LSM) and metformin in PCOS management.

Methods

This is a retrospective cohort study conducted among 130 PCOS patients who attended the outpatient department of the tertiary care hospital in central India from October 2019 to March 2020. The study describes the effect of a combined package of LSM (physical exercise and dietary changes) and metformin on anthropometric, clinical, and biochemical parameters at three and six months.

Results

Out of the total 130 women, 12 were lost to follow-up and were omitted from further analysis. At six months of the treatment package (LSM, metformin, and enhanced adherence counseling (EAC)), a significant decrease was seen in body mass index, blood sugar, follicle-stimulating hormone, luteinizing hormone, and insulin. Following the intervention, the menstruation cycle became regular in 91% of the women while volume, theca, and appearance of polycystic ovaries on ultrasound decreased in 86% of women. Insulin resistance (IR) and hyperinsulinemia are the major causes of pathophysiological changes associated with PCOS. Metformin along with LSM primarily acts by decreasing IR, while EAC ensures treatment compliance.

Conclusion

Metformin along with LSM in the form of calories restricted, high-protein diet, and physical activity reduce IR and hyperandrogenaemia, resulting in improved anthropometric, glycemic indices, hormonal profiles, and features of hyperandrogenaemia. The combined therapy is beneficial to 85-90% of women with PCOS.

## Introduction

Polycystic ovary syndrome (PCOS) is the commonest endocrine disorder in women of childbearing age [1]. In India, PCOS prevalence ranges from 3.7% to 22.5% [2-5]. Menstruation irregularities, clinical hyperandrogenism, and polycystic ovaries are characteristic of PCOS. PCOS patients have an increased risk of metabolic disorders, i.e., high body mass index (BMI), altered lipid profile, insulin resistance (IR), and type 2 diabetes mellitus. IR is prevailing in both thin and overweight women with PCOS [6]. For the diagnosis of PCOS, Rotterdam’s criteria are most commonly used [7,8]. Efficient management of PCOS may avoid the risk of cardiovascular complications associated with metabolic disorders in PCOS. Management of PCOS needs a sustained, multi-disciplinary expertise focusing on immediate clinical symptoms and addressing long-term concerns.

Yet, due to the complex nature of PCOS, individually tailored treatment options become a difficult clinical practice. For effective management of PCOS, both pharmacological and non-pharmacological treatment approaches are vital and have shown varied results [9-11]. The target of PCOS treatment strategies is to address the IR, oligoovulation, and hyperandrogenism. Lifestyle modification (LSM) plus metformin is an established management practice for PCOS patients and has shown good results [12].

The current study was aimed to assess the effects of metformin, LSM, and enhanced adherence counseling (EAC) on clinical symptoms, biochemical parameters, and anthropometric parameters in women with PCOS in a peri-urban medical college in central India.

## Materials and methods

The current study is a retrospective cohort analysis of 130 PCOS patients, who attended the gynecological outpatient department (OPD) of the institute (Datta Meghe Medical College, Datta Meghe Institute of Medical Sciences (Deemed to be University), Nagpur) from Oct 2019 to March 2020 and defines the effect of the combined package of LSM and metformin on clinical, anthropometric, and biochemical parameters [13]. Demography, clinical history, menstrual history, investigation findings, etc. of the study population were collected from OPD case records. The combined package of LSM, metformin (500 mg once a day to three times a day), and EAC was used for comprehensive management of PCOS patients.

LSM included dietary changes and physical activity. For obese adults (i.e., BMI > 23 kg/m^2^) and adolescents (> 97.5th percentile BMI for age), a calorie-deficit diet in consultation with a dietician was advised. Diet with low carbohydrate and fat and high protein (1 to 1.5 gm/kg body weight) results in a diet with 500 calories less than the daily requirement. For lean PCOS, dietary modifications were aimed at increasing the protein (1 to 1.5gm/kg body weight) content in the diet. Thirty minutes/day physical activity sessions were recommended. Women were motivated to complete at least five such sessions per week.

As IR is linked to androgen excess and consequential hyperinsulinemia, insulin sensitizers such as metformin (500 mg) were given OD to TDS, depending upon the clinical condition of the patient [14-16].

Dietary changes and exercise are the safest and sustainable interventions in management of PCOS. However, it is often difficult to make sustained changes in the lifestyle. To address this challenge, EAC, i.e., periodic counseling sessions (fortnightly in the first three months, followed by monthly sessions), was done from the beginning of the treatment along with dietary advice and continuous physical activities.

Periodic evaluation with respect to clinical improvement, i.e., Ferriman-Gallwey scale for hirsutism, menstrual cycles, and BMI was done at three and six months, while evaluation of changes in biochemical parameters and hormone levels (fasting blood glucose, two hours post-prandial sugar, fasting blood insulin, follicle-stimulating hormone (FSH), luteinizing hormone (LH)) was done at baseline and six months [17].

Statistical analysis 

The statistical analysis was carried out with IBM SPSS Statistics for Windows, Version 21 (Released 2012; IBM Corp., Armonk, New York, United States). While descriptive statistics were used to analyze continuous variables, which were then presented as the mean and the standard deviation (SD), frequency distributions and percentages were used to present categorical variables. For data presentation, tables and multiple bar charts were used. During analysis, appropriate tests of significance (T-test, Chi-square test and Fischer exact test) were used. If the P value was lower than 0.05, it indicated that there was a significant difference between the two groups, thus invalidating the null hypothesis.

## Results

Among the 130 patients, 12 patients did not come for follow-up and were excluded from further analysis. The mean age of women with PCOS was 24.2 years with a range of 16-37 years.

At six months of treatment package (LSM, metformin, and EAC), significant changes were observed in anthropometry and glycemic indices (Table 1). Weight decreased in 50% of women. Mean BMI reduced from baseline 28.4 kg/m^2^ to 23.9 kg/m^2^. Significant changes were also seen in the waist-hip ratio (WHR), fasting, and post-prandial blood sugar (P value <0.0001).

**Table 1:** Glycemic changes and anthropometric changes (n=118) BMI: Body mass index; WHR: waist-hip ratio; FBS: fasting blood sugar; PPBS: post-prandial blood sugar

Parameter		Baseline	Six Months after Treatment	P Value
BMI (kg/M2)	<18.5	9	11	<0.0001
	18.5-22.9	22	33	
	23-24.9	28	45	
	>25	59	29	
Mean BMI (Kg/m2)		28.4 ± 3.7	23.9 ± 3.2	< 0.0001
WHR		0.85 ± 0.18	0.63 ± 0.14	< 0.0001
FBS (mg/dl)		92.2 ± 8.8	85.5 ± 8.2	< 0.0001
2 h PPBS (mg/dl)		116.6 ± 19.6	104.8 ± 11.4	< 0.0001

With the improved insulin sensitivity following treatment, the mean blood insulin level decreased significantly from baseline 14.5 uIU/ml to 8.6 uIU/ml at six months (P value <0.0001). The LH/FSH ratio becomes non-significant in all women treated (Table 2).

**Table 2:** Hormone profile (n=118) LH: Luteinizing hormone; FSH: follicle-stimulating hormone

Parameter		Baseline	Six Months after Treatment	P Value
Mean FSH		5.2 ± 0.9	4.4 ± 0.8	< 0.0001
Mean LH		15.3 ± 3.2	7.3 ± 2.1	< 0.0001
LH/FSH Ratio	Significant (>3)	49	0	<0.0001
	Nonsignificant (<3)	69	118	
Insulin (uIU/ml)		14.5 ± 7.9	8.6 ± 2.14	< 0.0001

**Figure 1:**
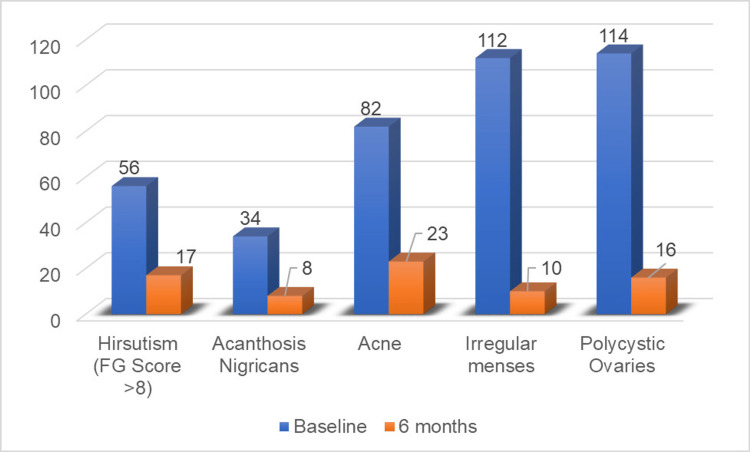
Effect of treatment on PCOS features PCOS: Polycystic ovary syndrome

Following the intervention, PCOS features improved in the majority of the women. The menstruation cycle became regular in 91% of the women. USG findings characteristic of PCOS became normal in 86% of women following treatment. Improvement was also observed in dermatological features of PCOS (Figure 1).

## Discussion

IR and hyperinsulinemia are the major causes of pathophysiological changes associated with PCOS, affecting 65-70% of women with PCOS [18]. Associated obesity further aggravates their IR. Insulin stimulates ovarian theca cells and androgen production and suppresses the production of sex hormone-binding globulin by the liver. The raised ovarian androgen disrupts follicle generation [19]. The resultant anovulation leads to unrestricted estrogen production and endometrial growth.

In the current study, after six months of metformin and LSM significant improvement was seen in all anthropometric, glycemic, and hormonal profiles and features of androgen excess (Tables 1, 2 and Figure 1). The role of metformin and LSM in PCOS management has been studied extensively either alone or in combination with other drugs, i.e., Clomiphene citrates, oral contraceptive pills, etc. Metformin along with LSM primarily acts by decreasing IR.

Weight reduction among obese/overweight PCOS women has been shown to regularize ovulation, balance androgen excess, and increase the rate of conception [20]. A 5-10% reduction in total body weight can decrease central fat by up to 30%, increase insulin sensitivity of peripheral tissues, reinstate ovulation, and be beneficial in metabolic disturbances [21].

The primary action of metformin appears to be increasing peripheral insulin sensitivity and inhibiting glucose production by the liver. It is associated with regularization of the menstruation cycle, enhanced ovulation, and decreased androgen level [22]. Metabolic benefits are greater in the presence of weight loss, and weight loss itself may be boosted in due to metformin [23]. Gastrointestinal side effects associated with metformin may be reduced by starting low doses of metformin (500 mg daily) and then slowly increasing the dose over four-six weeks. Sustained release preparations may further reduce the side effects of insulin.

The data of 608 PCOS women age ranging from 12 to 39 years across nine studies comparing LSM plus metformin use, LSM plus placebo, or metformin alone were analyzed. Most studies lasted six months (range three to 12 months). Patients treated with LSM plus metformin had a lower BMI than LSM plus placebo with a mean difference in the BMI of -0.73 kg/m² (n = 493; 95% CI, -1.14 to -0.32). LSM plus metformin arms had a statistically significantly higher number of menstrual cycles during six months compared to lifestyle plus placebo arms with a mean difference of 1.06 and P values of 0.006. Studies have shown diminished subcutaneous adipose tissue among subjects assigned lifestyle modification plus metformin compared to lifestyle plus placebo and had a mean difference of -92.49 cm² (P = 0.01) [10].

Sufficient evidence supports the favorable effect of metformin on hyperandrogenism in PCOS [22, 24-26]. Metformin may also reduce hirsutism through improvement in hyperandrogenaemia and probably by reducing circulating insulin levels. Studies have shown modest improvement in hair growth [27-30].

Limitations of the study 

Since this is a retrospective analysis of electronic health records, many important variables/information important to the management of PCOS could not be collected. The quality of adherence counseling could not be commented upon. Authors feel that a mixed method study (qualitative and quantitative) would have been better to study the impact of the management package. 

## Conclusions

Metformin along with lifestyle changes in the form of calorie restricted, high protein diet, and physical activity reduces iR and hyperandrogenaemia. The combined therapy shows benefit in > 85-90% women affected by PCOS. With the stoppage of metformin, these benefits may reverse, and lifestyle modification sustains the gain and reduces the dependence on pharmacotherapy. As lifestyle modifications are difficult to achieve and require constant motivation, periodic enhanced adherence counseling is required especially in the initial phase of treatment.

## References

[1] Norman RJ, Dewailly D, Legro RS, Hickey TE (2007). Polycystic ovary syndrome. Lancet.

[2] Gill H, Tiwari P, Dabadghao P (2012). Prevalence of polycystic ovary syndrome in young women from North India: a community-based study. Indian J Endocrinol Metab.

[3] Joshi B, Mukherjee S, Patil A, Purandare A, Chauhan S, Vaidya R (2014). A cross-sectional study of polycystic ovarian syndrome among adolescent and young girls in Mumbai, India. Indian J Endocrinol Metab.

[4] Vidya Bharathi R, Swetha S, Neerajaa J (2017). An epidemiological survey: Effect of predisposing factors for PCOS in Indian urban and rural population. Middle East Fertil Soc J.

[5] Nidhi R, Padmalatha V, Nagarathna R, Amritanshu R (2011). Prevalence of polycystic ovarian syndrome in Indian adolescents. J Pediatr Adolesc Gynecol.

[6] Ramezani Tehrani F, Amiri M, Behboudi-Gandevani S, Bidhendi-Yarandi R, Carmina E (2020). Cardiovascular events among reproductive and menopausal age women with polycystic ovary syndrome: a systematic review and meta-analysis. Gynecol Endocrinol.

[7] Fleischman A, Mansfield J (2005). Diagnosis and treatment of polycystic ovarian syndrome and insulin resistance. Pediatr Ann.

[8] Azziz R, Carmina E, Dewailly D (2009). The androgen excess and PCOS society criteria for the polycystic ovary syndrome: the complete task force report. Fertil Steril.

[9] Teede HJ, Misso ML, Costello MF (2018). Recommendations from the international evidence-based guideline for the assessment and management of polycystic ovary syndrome. Hum Reprod.

[10] Malik S, Jain K, Talwar P (2014). Management of polycystic ovary syndrome in India. Fertil Sci Res.

[11] Naderpoor N, Shorakae S, de Courten B, Misso ML, Moran LJ, Teede HJ (2016). Metformin and lifestyle modification in polycystic ovary syndrome: systematic review and meta-analysis. Hum Reprod Update.

[12] Abdolahian S, Tehrani FR, Amiri M (2020). Effect of lifestyle modifications on anthropometric, clinical, and biochemical parameters in adolescent girls with polycystic ovary syndrome: a systematic review and meta-analysis. BMC Endocr Disord.

[13] Jungari ML, Nair P, Gode S, Jaiswal A (2020). PCOS: clinical picture of PCOS patients in a peri urban tertiary care hospital of central India. JCR.

[14] Haas DA, Carr BR, Attia GR (2003). Effects of metformin on body mass index, menstrual cyclicity, and ovulation induction in women with polycystic ovary syndrome. Fertil Steril.

[15] Misra S, Parida N, Das S, Parija BS, Padhi M, Baig MA (2004). Effect of metformin in asian Indian women with polycystic ovarian syndrome. Metab Syndr Relat Disord.

[16] Attia GR, Rainey WE, Carr BR (2001). Metformin directly inhibits androgen production in human thecal cells. Fertil Steril.

[17] Lumezi BG, Berisha VL, Pupovci HL, Goçi A, Hajrushi AB (2018). Grading of hirsutism based on the Ferriman-Gallwey scoring system in Kosovar women. Postepy Dermatol Alergol.

[18] DeUgarte CM, Bartolucci AA, Azziz R (2005). Prevalence of insulin resistance in the polycystic ovary syndrome using the homeostasis model assessment. Fertil Steril.

[19] Jonard S, Dewailly D (2004). The follicular excess in polycystic ovaries, due to intra-ovarian hyperandrogenism, may be the main culprit for the follicular arrest. Hum Reprod Update.

[20] Norman RJ, Davies MJ, Lord J, Moran LJ (2002). The lifestyle modification in polycystic ovary syndrome. Trends Endocrinol Metab.

[21] Hoeger KM (2008). Exercise therapy in polycystic ovary syndrome. Semin Reprod Med.

[22] Nestler JE (2008). Metformin for the treatment of the polycystic ovary syndrome. N Engl J Med.

[23] Tan S, Hahn S, Benson S (2007). Metformin improves polycystic ovary syndrome symptoms irrespective of pre-treatment insulin resistance. Eur J Endocrinol.

[24] Legro RS, Barnhart HX, Schlaff WD (2007). Clomiphene, metformin, or both for infertility in the polycystic ovary syndrome. N Engl J Med.

[25] Nawrocka J, Starczewski A (2007). Effects of metformin treatment in women with polycystic ovary syndrome depends on insulin resistance. Gynecol Endocrinol.

[26] Marcondes JA, Yamashita SA, Maciel GA, Baracat EC, Halpern A (2007). Metformin in normal-weight hirsute women with polycystic ovary syndrome with normal insulin sensitivity. Gynecol Endocrinol.

[27] Barbieri RL (2007). Clomiphene versus metformin for ovulation induction in polycystic ovary syndrome: the winner is ... J Clin Endocrinol Metab.

[28] Moghetti P, Castello R, Negri C (2000). Metformin effects on clinical features, endocrine and metabolic profiles, and insulin sensitivity in polycystic ovary syndrome: a randomized, double-blind, placebo-controlled 6-month trial, followed by open, long-term clinical evaluation.. J Clin Endocrinol Metab.

[29] Kolodziejczyk B, Duleba AJ, Spaczynski RZ, Pawelczyk L (2000). Metformin therapy decreases hyperandrogenism and hyperinsulinemia in women with polycystic ovary syndrome. Fertil Steril.

[30] Kelly CJ, Gordon D (2002). The effect of metformin on hirsutism in polycystic ovary syndrome. Eur J Endocrinol.

